# 4-[(*Z*)-(2-Eth­oxy-4-oxochroman-3-yl­idene)methyl­amino]benzene­sulfonamide monohydrate

**DOI:** 10.1107/S1600536809026154

**Published:** 2009-07-11

**Authors:** Mariya-al-Rashida, M. Nawaz Tahir, Saeed Ahmad Nagra, Muhammed Imran, Javed Iqbal

**Affiliations:** a Institute of Chemistry, University of the Punjab, Lahore, Pakistan; b Department of Physics, University of Sargodha, Sargodha, Pakistan

## Abstract

In the mol­ecule of the title compound, C_18_H_18_N_2_O_5_S·H_2_O, the heterocyclic ring adopts a twisted conformation, while the aromatic rings are oriented at a dihedral angle of 45.46 (3)°. Intra­molecular C—H⋯O and N—H⋯O inter­actions result in the formations of planar five- and six-membered rings. In the crystal structure, N—H⋯O hydrogen bonds link the NH_2_ and SO_2_ groups through *R*_2_^2^(8) ring motifs, while C—H⋯O and N—H⋯O hydrogen bonds result in the formation of *R*_2_^1^(7) ring motifs. N—H⋯O and O—H⋯O hydrogen bonds link the uncoordinated water mol­ecules, forming a polymeric network. A weak C—H⋯π inter­action is also present.

## Related literature

For related structures, see: Al-Zaydi *et al.* (2007 ▶); Chohan *et al.* (2008 ▶, 2009 ▶). For ring puckering parameters, see: Cremer & Pople (1975 ▶). For ring motifs, see: Bernstein *et al.* (1995 ▶). For bond-length data, see: Allen *et al.* (1987 ▶).
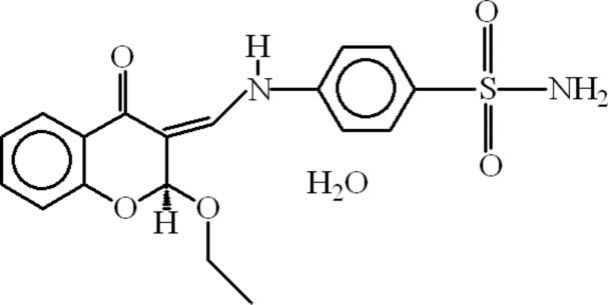


## Experimental

### 

#### Crystal data

C_18_H_18_N_2_O_5_S·H_2_O
*M* *_r_* = 392.42
Triclinic, 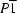

*a* = 8.2727 (6) Å
*b* = 10.0166 (8) Å
*c* = 11.5830 (9) Å
α = 102.480 (5)°
β = 97.049 (4)°
γ = 96.731 (4)°
*V* = 919.77 (12) Å^3^
*Z* = 2
Mo *K*α radiation
μ = 0.21 mm^−1^
*T* = 296 K
0.28 × 0.10 × 0.09 mm

#### Data collection

Bruker Kappa APEXII CCD diffractometer
Absorption correction: multi-scan (*SADABS*; Bruker, 2005 ▶) *T*_min_ = 0.973, *T*_max_ = 0.982
18417 measured reflections
4729 independent reflections
2022 reflections with *I* > 2σ(*I*)
*R*_int_ = 0.070

#### Refinement

*R*[*F* ^2^ > 2σ(*F* ^2^)] = 0.060
*wR*(*F* ^2^) = 0.125
*S* = 1.00
4729 reflections
260 parameters
H atoms treated by a mixture of independent and constrained refinement
Δρ_max_ = 0.25 e Å^−3^
Δρ_min_ = −0.22 e Å^−3^

### 

Data collection: *APEX2* (Bruker, 2007 ▶); cell refinement: *SAINT* (Bruker, 2007 ▶); data reduction: *SAINT*; program(s) used to solve structure: *SHELXS97* (Sheldrick, 2008 ▶); program(s) used to refine structure: *SHELXL97* (Sheldrick, 2008 ▶); molecular graphics: *ORTEP-3 for Windows* (Farrugia, 1997 ▶) and *PLATON* (Spek, 2009 ▶); software used to prepare material for publication: *WinGX* (Farrugia, 1999 ▶) and *PLATON*.

## Supplementary Material

Crystal structure: contains datablocks global, I. DOI: 10.1107/S1600536809026154/hk2734sup1.cif

Structure factors: contains datablocks I. DOI: 10.1107/S1600536809026154/hk2734Isup2.hkl

Additional supplementary materials:  crystallographic information; 3D view; checkCIF report

## Figures and Tables

**Table 1:** Hydrogen-bond geometry (Å, °)

*D*—H⋯*A*	*D*—H	H⋯*A*	*D*⋯*A*	*D*—H⋯*A*
N1—H1⋯O4	0.86	2.04	2.696 (3)	132
N2—H21⋯O6^i^	0.91 (3)	1.94 (3)	2.838 (4)	169 (3)
N2—H22⋯O2^ii^	0.85 (3)	2.22 (3)	3.027 (3)	159 (3)
O6—H61⋯O5	0.90 (4)	2.10 (4)	2.999 (3)	176.7 (16)
O6—H62⋯O4^iii^	0.92 (3)	1.90 (3)	2.784 (3)	161 (3)
C2—H2⋯O2^iv^	0.93	2.42	3.300 (4)	157
C9—H9⋯O1^v^	1.04 (3)	2.54 (3)	3.472 (3)	149 (2)
C13—H13⋯O2^ii^	0.93	2.54	3.418 (3)	158
C15—H15⋯O1	0.93	2.50	2.884 (3)	105
C16—H16⋯*Cg*1^vi^	0.93	2.95	3.565 (3)	125

Symmetry codes: (i) 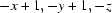; (ii) 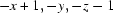; (iii) 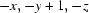; (iv) 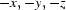; (v) 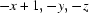; (vi) 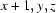. *Cg*1 is the centroid of the C1–C6 ring.

**Fig. 1.**
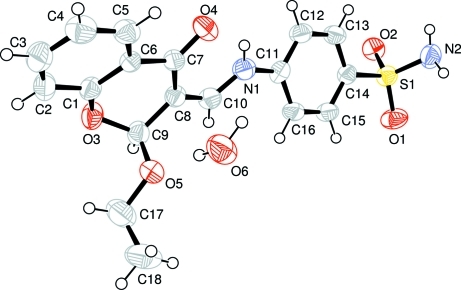
The molecular structure of the title molecule, with the atom-numbering scheme. Displacement ellipsoids are drawn at the 50% probability level.

**Fig. 2.**
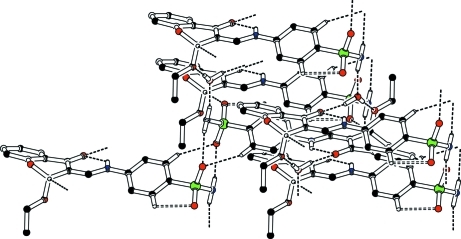
A partial packing diagram of the title compound. Hydrogen bonds are shown as dashed lines.

## supplementary crystallographic information

### Comment

Sulfonamides have wide range of applications in medicinal chemistry. Keeping in wiew the importance of sulfonamide derivatives, we have synthesized the title compound, (I). We report herein its crystal structure.

The crystal structures of 4-[(*E*)-(5-chloro-2-hydroxybenzylidene)amino] benzenesulfonamide, (II) (Chohan *et al.*, 2009) and 4-{2-[(5-chloro-2 -hydroxybenzylidene)amino]ethyl}benzenesulfonamide, (III) (Chohan *et al.*, 2008) have been published, which contain the benzenesulfonamide. The crystal structure of 3-(4-chlorophenylhydrazono)-2-ethoxychroman-4-one, (IV) (Al-Zaydi *et al.*, 2007) has also been published, which contains the common moiety of (I) other than benzenesulfonamide.

In the molecule of the title compound (Fig. 1), the bond lengths (Allen *et al.*,  1987) and angles are within normal ranges. Ring B (O3/C1/C6-C9) is not planar, having total puckering amplitude, Q_T_, of 0.444 (3) Å and twisted conformation [φ = 84.93 (3) and θ = 70.07 (3) °] (Cremer & Pople, 1975). Rings A (C1-C6) and C (C11-C16) are, of course, planar, and they are oriented at a dihedral angle of A/C = 45.46 (3)°. Intramolecular C-H···O and N-H···O interactions (Table 1) result in the formations of planar five- and six-membered rings D (S1/O1/C14/C15/H15) and E (O4/N1/C7/C8/C10/H1).

In the crystal structure, N-H···O hydrogen bonds link the NH_2_ and SO_2_ groups through *R*_2_^2^(8) ring motifs, while C-H···O and N-H···O hydrogen bonds (Table 1) result in the formations of *R*_2_^1^(7) ring motifs (Bernstein *et al.*, 1995). On the other hand, N-H···O and O-H···O hydrogen bonds (Table 1) link the lattice water molecules to form a polymeric network (Fig. 2), in which they may be effective in the stabilization of the structure. There also exists a weak C—H···π interaction.

### Experimental

3-Formylchromone (0.174 g, 1 mmol) in ethanol (5-7 ml) was stirred with heating until dissolved, then catalytic amount of *p*-toluenesulfonic acid was added, followed by 4-aminobenzenesulfonamide (0.172 g, 1 mmol) in equal amount of ethanol. Reaction mixture was refluxed with stirring for 4 h. The clear yellow solution was kept overnight and solvent was evaporated to yield bright yellow crystalline solid. Product was recrystallized from a mixture of ethanol and acetone (1:1) to yield fine transparent yellow needles.

### Refinement

H atoms (for NH_2_, OH_2_ and methine) were located in a difference Fourier map and their coordinates were refined. The remaining H atoms were positioned geometrically with N-H = 0.86 Å (for NH) and C-H = 0.93, 0.97 and 0.96 Å for aromatic, methylene and methyl H atoms, respectively and constrained to ride on their parent atoms, with *U*_iso_(H) = *xU*_eq_(C,*N*,*O*), where *x* = 1.5 for methyl H and x = 1.2 for all other H atoms.

### Crystal data

**Table:**

C_18_H_18_N_2_O_5_S·H_2_O	*Z* = 2
*M**_r_* = 392.42	*F*(000) = 412
Triclinic, *P*1	*D*_x_ = 1.417 Mg m^−^^3^
Hall symbol: -P 1	Mo *K*α radiation, λ = 0.71073 Å
*a* = 8.2727 (6) Å	Cell parameters from 4729 reflections
*b* = 10.0166 (8) Å	θ = 2.4–28.8°
*c* = 11.5830 (9) Å	µ = 0.21 mm^−^^1^
α = 102.480 (5)°	*T* = 296 K
β = 97.049 (4)°	Needle, yellow
γ = 96.731 (4)°	0.28 × 0.10 × 0.09 mm
*V* = 919.77 (12) Å^3^	

### Data collection

**Table:**

Bruker Kappa APEXII CCD diffractometer	4729 independent reflections
Radiation source: fine-focus sealed tube	2022 reflections with *I* > 2σ(*I*)
graphite	*R*_int_ = 0.070
Detector resolution: 7.40 pixels mm^-1^	θ_max_ = 28.8°, θ_min_ = 2.4°
ω scans	*h* = −11→7
Absorption correction: multi-scan (*SADABS*; Bruker, 2005)	*k* = −13→13
*T*_min_ = 0.973, *T*_max_ = 0.982	*l* = −15→15
18417 measured reflections	

### Refinement

**Table:**

Refinement on *F*^2^	Primary atom site location: structure-invariant direct methods
Least-squares matrix: full	Secondary atom site location: difference Fourier map
*R*[*F*^2^ > 2σ(*F*^2^)] = 0.060	Hydrogen site location: inferred from neighbouring sites
*wR*(*F*^2^) = 0.125	H atoms treated by a mixture of independent and constrained refinement
*S* = 1.00	*w* = 1/[σ^2^(*F*_o_^2^) + (0.0427*P*)^2^] where *P* = (*F*_o_^2^ + 2*F*_c_^2^)/3
4729 reflections	(Δ/σ)_max_ < 0.001
260 parameters	Δρ_max_ = 0.25 e Å^−^^3^
0 restraints	Δρ_min_ = −0.21 e Å^−^^3^

### Special details

**Table:**

Geometry. Bond distances, angles *etc*. have been calculated using the rounded fractional coordinates. All su's are estimated from the variances of the (full) variance-covariance matrix. The cell e.s.d.'s are taken into account in the estimation of distances, angles and torsion angles
Refinement. Refinement of *F*^2^ against ALL reflections. The weighted *R*-factor *wR* and goodness of fit *S* are based on *F*^2^, conventional *R*-factors *R* are based on *F*, with *F* set to zero for negative *F*^2^. The threshold expression of *F*^2^ > σ(*F*^2^) is used only for calculating *R*-factors(gt) *etc*. and is not relevant to the choice of reflections for refinement. *R*-factors based on *F*^2^ are statistically about twice as large as those based on *F*, and *R*- factors based on ALL data will be even larger.

### Fractional atomic coordinates and isotropic or equivalent isotropic displacement parameters (Å^2^)

**Table:**

	*x*	*y*	*z*	*U*_iso_*/*U*_eq_	
S1	0.65357 (9)	0.03978 (8)	−0.32059 (7)	0.0444 (3)	
O1	0.7917 (2)	0.0360 (2)	−0.23543 (17)	0.0608 (8)	
O2	0.5862 (2)	−0.08348 (18)	−0.41008 (16)	0.0501 (7)	
O3	−0.2219 (2)	0.19797 (19)	0.24854 (17)	0.0513 (7)	
O4	−0.1312 (2)	0.3631 (2)	−0.03129 (17)	0.0580 (8)	
O5	0.0431 (2)	0.3149 (2)	0.31661 (16)	0.0510 (7)	
O6	0.1102 (3)	0.5917 (2)	0.2582 (2)	0.0705 (9)	
N1	0.1144 (3)	0.2091 (2)	−0.0546 (2)	0.0478 (9)	
N2	0.7070 (3)	0.1526 (3)	−0.3923 (3)	0.0559 (10)	
C1	−0.3062 (3)	0.3009 (3)	0.2233 (3)	0.0412 (10)	
C2	−0.4381 (3)	0.3273 (3)	0.2838 (3)	0.0558 (11)	
C3	−0.5269 (4)	0.4275 (3)	0.2598 (3)	0.0628 (11)	
C4	−0.4871 (4)	0.5029 (3)	0.1782 (3)	0.0588 (12)	
C5	−0.3605 (3)	0.4734 (3)	0.1166 (3)	0.0491 (11)	
C6	−0.2684 (3)	0.3703 (3)	0.1377 (2)	0.0376 (9)	
C7	−0.1412 (3)	0.3271 (3)	0.0643 (2)	0.0403 (9)	
C8	−0.0367 (3)	0.2398 (3)	0.1090 (2)	0.0405 (9)	
C9	−0.0540 (3)	0.2094 (3)	0.2276 (3)	0.0470 (11)	
C10	0.0843 (3)	0.1890 (3)	0.0508 (3)	0.0469 (11)	
C11	0.2442 (3)	0.1665 (3)	−0.1153 (3)	0.0424 (10)	
C12	0.2237 (3)	0.1498 (3)	−0.2378 (3)	0.0479 (11)	
C13	0.3481 (3)	0.1123 (3)	−0.3005 (2)	0.0463 (10)	
C14	0.4946 (3)	0.0904 (3)	−0.2408 (2)	0.0392 (9)	
C15	0.5153 (3)	0.1090 (3)	−0.1186 (3)	0.0455 (10)	
C16	0.3895 (3)	0.1473 (3)	−0.0555 (2)	0.0471 (10)	
C17	0.0555 (4)	0.2828 (4)	0.4337 (3)	0.0756 (16)	
C18	0.1854 (4)	0.3812 (4)	0.5152 (3)	0.0916 (16)	
H1	0.04755	0.25264	−0.08969	0.0574*	
H2	−0.46546	0.27765	0.33962	0.0668*	
H3	−0.61609	0.44513	0.29940	0.0754*	
H4	−0.54606	0.57326	0.16535	0.0707*	
H5	−0.33511	0.52237	0.05987	0.0590*	
H9	−0.013 (3)	0.120 (3)	0.243 (2)	0.0563*	
H10	0.15063	0.13653	0.08753	0.0562*	
H12	0.12514	0.16395	−0.27815	0.0573*	
H13	0.33404	0.10157	−0.38301	0.0557*	
H15	0.61413	0.09592	−0.07795	0.0545*	
H16	0.40398	0.15997	0.02722	0.0565*	
H17A	0.08048	0.18955	0.42767	0.0909*	
H17B	−0.04851	0.28840	0.46382	0.0909*	
H18A	0.16603	0.47367	0.51433	0.1377*	
H18B	0.28992	0.36745	0.49030	0.1377*	
H18C	0.18604	0.36752	0.59477	0.1377*	
H21	0.757 (3)	0.234 (3)	−0.342 (3)	0.0671*	
H22	0.634 (4)	0.155 (3)	−0.450 (3)	0.0671*	
H61	0.086 (4)	0.508 (4)	0.274 (3)	0.0846*	
H62	0.110 (4)	0.586 (3)	0.178 (3)	0.0846*	

### Atomic displacement parameters (Å^2^)

**Table:**

	*U*^11^	*U*^22^	*U*^33^	*U*^12^	*U*^13^	*U*^23^
S1	0.0396 (4)	0.0481 (5)	0.0453 (5)	0.0093 (3)	0.0075 (3)	0.0083 (4)
O1	0.0433 (11)	0.0813 (16)	0.0562 (14)	0.0244 (10)	0.0001 (10)	0.0088 (11)
O2	0.0598 (12)	0.0394 (12)	0.0470 (12)	0.0060 (10)	0.0096 (10)	0.0015 (10)
O3	0.0436 (11)	0.0510 (13)	0.0676 (14)	0.0071 (10)	0.0156 (10)	0.0278 (11)
O4	0.0622 (13)	0.0742 (15)	0.0450 (13)	0.0114 (11)	0.0120 (10)	0.0273 (12)
O5	0.0523 (12)	0.0600 (14)	0.0410 (12)	0.0048 (10)	0.0064 (10)	0.0149 (11)
O6	0.0939 (17)	0.0584 (15)	0.0519 (14)	−0.0130 (13)	0.0058 (13)	0.0132 (13)
N1	0.0435 (14)	0.0551 (16)	0.0440 (15)	0.0087 (12)	0.0052 (12)	0.0098 (13)
N2	0.0537 (17)	0.0521 (17)	0.0577 (19)	−0.0052 (14)	0.0098 (13)	0.0099 (15)
C1	0.0357 (15)	0.0402 (17)	0.0482 (18)	0.0026 (14)	0.0053 (14)	0.0140 (15)
C2	0.0441 (18)	0.069 (2)	0.063 (2)	0.0092 (17)	0.0175 (16)	0.0283 (18)
C3	0.0491 (19)	0.079 (2)	0.067 (2)	0.0201 (19)	0.0192 (17)	0.020 (2)
C4	0.061 (2)	0.058 (2)	0.060 (2)	0.0250 (17)	0.0078 (18)	0.0118 (18)
C5	0.0515 (18)	0.0467 (19)	0.0511 (19)	0.0082 (16)	0.0034 (16)	0.0176 (16)
C6	0.0356 (15)	0.0384 (16)	0.0357 (16)	−0.0013 (13)	0.0018 (13)	0.0079 (14)
C7	0.0411 (16)	0.0420 (17)	0.0328 (16)	−0.0036 (14)	0.0037 (14)	0.0045 (14)
C8	0.0365 (15)	0.0442 (17)	0.0399 (17)	0.0053 (14)	0.0063 (14)	0.0079 (14)
C9	0.0425 (17)	0.052 (2)	0.050 (2)	0.0096 (16)	0.0121 (15)	0.0154 (17)
C10	0.0437 (17)	0.0494 (19)	0.0453 (19)	0.0031 (15)	0.0011 (15)	0.0114 (15)
C11	0.0353 (16)	0.0406 (17)	0.0481 (19)	0.0032 (14)	0.0076 (15)	0.0043 (14)
C12	0.0400 (16)	0.059 (2)	0.0435 (19)	0.0105 (15)	−0.0002 (15)	0.0115 (16)
C13	0.0452 (17)	0.059 (2)	0.0350 (16)	0.0122 (15)	0.0045 (14)	0.0102 (15)
C14	0.0375 (15)	0.0382 (16)	0.0401 (17)	0.0032 (13)	0.0050 (13)	0.0073 (14)
C15	0.0420 (16)	0.0506 (19)	0.0392 (18)	0.0047 (14)	−0.0021 (14)	0.0065 (15)
C16	0.0441 (17)	0.062 (2)	0.0294 (15)	0.0028 (15)	0.0008 (14)	0.0035 (14)
C17	0.087 (3)	0.094 (3)	0.053 (2)	0.018 (2)	0.015 (2)	0.028 (2)
C18	0.099 (3)	0.116 (3)	0.051 (2)	0.013 (3)	−0.010 (2)	0.014 (2)

### Geometric parameters (Å, °)

**Table:**

S1—O1	1.425 (2)	C8—C10	1.361 (4)
S1—O2	1.433 (2)	C8—C9	1.490 (4)
S1—N2	1.594 (3)	C11—C16	1.367 (4)
S1—C14	1.759 (3)	C11—C12	1.379 (5)
O3—C1	1.372 (3)	C12—C13	1.373 (4)
O3—C9	1.435 (3)	C13—C14	1.385 (4)
O4—C7	1.245 (3)	C14—C15	1.374 (4)
O5—C9	1.397 (4)	C15—C16	1.387 (4)
O5—C17	1.454 (4)	C17—C18	1.455 (5)
O6—H62	0.92 (3)	C2—H2	0.9300
O6—H61	0.90 (4)	C3—H3	0.9300
N1—C11	1.412 (4)	C4—H4	0.9300
N1—C10	1.327 (4)	C5—H5	0.9300
N1—H1	0.8600	C9—H9	1.04 (3)
N2—H21	0.91 (3)	C10—H10	0.9300
N2—H22	0.85 (3)	C12—H12	0.9300
C1—C6	1.374 (4)	C13—H13	0.9300
C1—C2	1.388 (4)	C15—H15	0.9300
C2—C3	1.367 (4)	C16—H16	0.9300
C3—C4	1.378 (5)	C17—H17B	0.9700
C4—C5	1.363 (4)	C17—H17A	0.9700
C5—C6	1.398 (4)	C18—H18C	0.9600
C6—C7	1.478 (3)	C18—H18A	0.9600
C7—C8	1.429 (4)	C18—H18B	0.9600
			
O1···N1^i^	3.239 (3)	C7···H1	2.5600
O2···C13^ii^	3.418 (3)	C7···H61	2.98 (4)
O2···N2^ii^	3.027 (3)	C7···H62	3.06 (3)
O2···C2^iii^	3.300 (4)	C8···H61	2.93 (4)
O3···C13^iii^	3.364 (4)	C9···H61	2.99 (4)
O4···O4^iv^	3.181 (3)	C10···H16	2.7300
O4···O6^iv^	2.784 (3)	C11···H5^iv^	3.0300
O4···N1	2.696 (3)	C12···H9^iii^	3.02 (3)
O5···C6	3.281 (3)	C16···H10	2.7400
O5···O6	2.999 (3)	H1···O4	2.0400
O6···C7	3.374 (3)	H1···O1^vii^	2.9200
O6···O5	2.999 (3)	H1···C7	2.5600
O6···N2^v^	2.838 (4)	H1···H12	2.3700
O6···O4^iv^	2.784 (3)	H1···H62^iv^	2.5000
O1···H15	2.5000	H2···O2^iii^	2.4200
O1···H9^vi^	2.54 (3)	H3···O6^vii^	2.8900
O1···H1^i^	2.9200	H4···H5^ix^	2.6000
O1···H10^vi^	2.7200	H5···C5^ix^	3.0500
O2···H22^ii^	2.22 (3)	H5···C4^ix^	2.9100
O2···H13^ii^	2.5400	H5···O4	2.6200
O2···H2^iii^	2.4200	H5···C11^iv^	3.0300
O3···H17B	2.6300	H5···H4^ix^	2.6000
O4···H5	2.6200	H9···H10	2.4000
O4···H62^iv^	1.90 (3)	H9···C12^iii^	3.02 (3)
O4···H1	2.0400	H9···H17A	2.1200
O5···H61	2.10 (4)	H9···O1^vi^	2.54 (3)
O6···H3^i^	2.8900	H10···C16	2.7400
O6···H21^v^	1.94 (3)	H10···H16	2.2900
N1···O1^vii^	3.239 (3)	H10···O1^vi^	2.7200
N1···O4	2.696 (3)	H10···H9	2.4000
N2···O6^v^	2.838 (4)	H12···H1	2.3700
N2···O2^ii^	3.027 (3)	H13···O2^ii^	2.5400
C2···C18^viii^	3.571 (5)	H15···O1	2.5000
C2···O2^iii^	3.300 (4)	H15···C7^i^	2.9300
C4···C5^ix^	3.560 (5)	H16···C1^i^	3.0600
C5···C5^ix^	3.505 (4)	H16···C10	2.7300
C5···C4^ix^	3.560 (5)	H16···H10	2.2900
C6···C16^vii^	3.579 (4)	H17A···H9	2.1200
C6···O5	3.281 (3)	H17B···O3	2.6300
C7···O6	3.374 (3)	H21···H62^v^	2.37 (5)
C7···C15^vii^	3.522 (4)	H21···O6^v^	1.94 (3)
C13···O3^iii^	3.364 (4)	H22···O2^ii^	2.22 (3)
C13···O2^ii^	3.418 (3)	H61···O5	2.10 (4)
C15···C7^i^	3.522 (4)	H61···C7	2.98 (4)
C16···C6^i^	3.579 (4)	H61···C8	2.93 (4)
C18···C2^viii^	3.571 (5)	H61···C9	2.99 (4)
C1···H16^vii^	3.0600	H62···C7	3.06 (3)
C4···H5^ix^	2.9100	H62···O4^iv^	1.90 (3)
C5···H5^ix^	3.0500	H62···H1^iv^	2.5000
C7···H15^vii^	2.9300	H62···H21^v^	2.37 (5)
			
O1—S1—O2	118.76 (12)	C12—C13—C14	120.0 (2)
O1—S1—N2	107.72 (14)	S1—C14—C13	120.33 (17)
O1—S1—C14	107.54 (11)	S1—C14—C15	120.1 (2)
O2—S1—N2	105.54 (15)	C13—C14—C15	119.5 (2)
O2—S1—C14	107.37 (12)	C14—C15—C16	120.3 (2)
N2—S1—C14	109.74 (15)	C11—C16—C15	119.8 (2)
C1—O3—C9	115.5 (2)	O5—C17—C18	109.0 (3)
C9—O5—C17	112.4 (2)	C1—C2—H2	121.00
H61—O6—H62	112 (3)	C3—C2—H2	121.00
C10—N1—C11	127.0 (2)	C4—C3—H3	119.00
C10—N1—H1	116.00	C2—C3—H3	119.00
C11—N1—H1	117.00	C5—C4—H4	120.00
S1—N2—H22	113 (2)	C3—C4—H4	120.00
H21—N2—H22	119 (3)	C4—C5—H5	120.00
S1—N2—H21	112 (2)	C6—C5—H5	120.00
O3—C1—C2	117.0 (3)	O3—C9—H9	105.4 (14)
O3—C1—C6	122.0 (2)	O5—C9—H9	104.0 (14)
C2—C1—C6	121.0 (3)	C8—C9—H9	116.5 (13)
C1—C2—C3	118.8 (3)	C8—C10—H10	118.00
C2—C3—C4	121.4 (3)	N1—C10—H10	118.00
C3—C4—C5	119.4 (3)	C11—C12—H12	120.00
C4—C5—C6	120.7 (3)	C13—C12—H12	120.00
C1—C6—C7	119.4 (3)	C12—C13—H13	120.00
C1—C6—C5	118.7 (2)	C14—C13—H13	120.00
C5—C6—C7	121.8 (2)	C16—C15—H15	120.00
O4—C7—C6	120.9 (2)	C14—C15—H15	120.00
O4—C7—C8	123.5 (2)	C15—C16—H16	120.00
C6—C7—C8	115.5 (2)	C11—C16—H16	120.00
C7—C8—C10	122.8 (2)	O5—C17—H17A	110.00
C7—C8—C9	118.8 (2)	C18—C17—H17A	110.00
C9—C8—C10	118.3 (3)	C18—C17—H17B	110.00
O3—C9—O5	109.9 (2)	O5—C17—H17B	110.00
O5—C9—C8	108.6 (2)	H17A—C17—H17B	108.00
O3—C9—C8	112.1 (2)	C17—C18—H18B	109.00
N1—C10—C8	124.6 (3)	C17—C18—H18C	110.00
C12—C11—C16	120.1 (2)	C17—C18—H18A	109.00
N1—C11—C16	121.8 (3)	H18A—C18—H18C	109.00
N1—C11—C12	118.1 (2)	H18B—C18—H18C	109.00
C11—C12—C13	120.3 (2)	H18A—C18—H18B	109.00
			
O1—S1—C14—C13	175.5 (2)	C4—C5—C6—C7	174.1 (3)
O1—S1—C14—C15	−4.3 (3)	C1—C6—C7—O4	162.4 (3)
O2—S1—C14—C13	−55.6 (3)	C1—C6—C7—C8	−15.9 (4)
O2—S1—C14—C15	124.6 (2)	C5—C6—C7—O4	−12.7 (4)
N2—S1—C14—C13	58.6 (3)	C5—C6—C7—C8	169.0 (3)
N2—S1—C14—C15	−121.2 (3)	O4—C7—C8—C9	176.8 (3)
C9—O3—C1—C2	−154.9 (3)	O4—C7—C8—C10	0.5 (4)
C9—O3—C1—C6	28.5 (4)	C6—C7—C8—C9	−4.9 (4)
C1—O3—C9—O5	73.9 (3)	C6—C7—C8—C10	178.7 (3)
C1—O3—C9—C8	−47.0 (3)	C7—C8—C9—O3	35.4 (4)
C17—O5—C9—O3	66.6 (3)	C7—C8—C9—O5	−86.2 (3)
C17—O5—C9—C8	−170.5 (2)	C10—C8—C9—O3	−148.0 (3)
C9—O5—C17—C18	167.6 (3)	C10—C8—C9—O5	90.4 (3)
C11—N1—C10—C8	175.7 (3)	C7—C8—C10—N1	−3.0 (5)
C10—N1—C11—C12	156.4 (3)	C9—C8—C10—N1	−179.4 (3)
C10—N1—C11—C16	−26.2 (4)	N1—C11—C12—C13	178.2 (3)
O3—C1—C2—C3	−179.0 (3)	C16—C11—C12—C13	0.8 (5)
C6—C1—C2—C3	−2.3 (5)	N1—C11—C16—C15	−178.3 (3)
O3—C1—C6—C5	179.6 (3)	C12—C11—C16—C15	−1.0 (5)
O3—C1—C6—C7	4.4 (4)	C11—C12—C13—C14	0.3 (5)
C2—C1—C6—C5	3.1 (4)	C12—C13—C14—S1	179.0 (2)
C2—C1—C6—C7	−172.2 (3)	C12—C13—C14—C15	−1.2 (5)
C1—C2—C3—C4	−0.6 (5)	S1—C14—C15—C16	−179.2 (2)
C2—C3—C4—C5	2.6 (5)	C13—C14—C15—C16	1.0 (5)
C3—C4—C5—C6	−1.7 (5)	C14—C15—C16—C11	0.1 (5)
C4—C5—C6—C1	−1.1 (4)		

Symmetry codes: (i) *x*+1, *y*, *z*; (ii) −*x*+1, −*y*, −*z*−1; (iii) −*x*, −*y*, −*z*; (iv) −*x*, −*y*+1, −*z*; (v) −*x*+1, −*y*+1, −*z*; (vi) −*x*+1, −*y*, −*z*; (vii) *x*−1, *y*, *z*; (viii) −*x*, −*y*+1, −*z*+1; (ix) −*x*−1, −*y*+1, −*z*.

### Hydrogen-bond geometry (Å, °)

**Table:**

*D*—H···*A*	*D*—H	H···*A*	*D*···*A*	*D*—H···*A*
N1—H1···O4	0.86	2.04	2.696 (3)	132
N2—H21···O6^v^	0.91 (3)	1.94 (3)	2.838 (4)	169 (3)
N2—H22···O2^ii^	0.85 (3)	2.22 (3)	3.027 (3)	159 (3)
O6—H61···O5	0.90 (4)	2.10 (4)	2.999 (3)	176.7 (16)
O6—H62···O4^iv^	0.92 (3)	1.90 (3)	2.784 (3)	161 (3)
C2—H2···O2^iii^	0.93	2.42	3.300 (4)	157
C9—H9···O1^vi^	1.04 (3)	2.54 (3)	3.472 (3)	149 (2)
C13—H13···O2^ii^	0.93	2.54	3.418 (3)	158
C15—H15···O1	0.93	2.50	2.884 (3)	105
C16—H16···Cg1^i^	0.93	2.95	3.565 (3)	125

Symmetry codes: (v) −*x*+1, −*y*+1, −*z*; (ii) −*x*+1, −*y*, −*z*−1; (iv) −*x*, −*y*+1, −*z*; (iii) −*x*, −*y*, −*z*; (vi) −*x*+1, −*y*, −*z*; (i) *x*+1, *y*, *z*.
